# Real‑World Clinical Characterization of Major Depressive Disorder and Treatment‑Resistant Depression Supported by Natural Language Processing: Multicenter Observational Study From the MOOD Project

**DOI:** 10.2196/86448

**Published:** 2026-05-22

**Authors:** Dieter Zeeuws, Katrien Bernagie, Geert De Bruecker, Isabel Claeys, Charlotte Evenepoel, Fabienne Ver Donck, David Smeets, Elke Peeters

**Affiliations:** 1 Department of Psychiatry Universitair Ziekenhuis Brussel Jette, Brussels Capital Belgium; 2 Neuroprotection and Neuromodulation Research Group (NEUR), Center for Neurosciences (C4N) Vrije Universiteit Brussel Brussels, Brussels Capital Belgium; 3 Psychiatric Center Ariadne Lede Belgium; 4 AZ Maria Middelares Ghent Belgium; 5 LynxCare Clinical Informatics Leuven Belgium; 6 Department External and Government Affairs Johnson & Johnson Beerse Belgium; 7 Department Medical Affairs Johnson & Johnson Beerse Belgium

**Keywords:** major depressive disorder, treatment-resistant depression, real-world evidence, natural language processing, symptom clusters, longitudinal data, clinical practice

## Abstract

**Background:**

Major depressive disorder (MDD) and treatment-resistant depression (TRD) are heterogeneous conditions in which key clinical details are split across structured fields and free-text notes in electronic health records (EHRs), constraining population-level insight and timely audit of care quality.

**Objective:**

This study aims to present a clinician-oriented, artificial intelligence-supported real-world evidence (RWE) methodology integrating structured and unstructured EHR data to profile MDD and TRD, and report comorbidity patterns from a 2-site pilot. This analysis reports the first objective of the MOOD project, which is to characterize the real‑world clinical and disease severity profile of patients with MDD and treatment‑resistant depression, providing a necessary foundation for subsequent evaluations of treatment patterns and outcomes.

**Methods:**

We conducted a retrospective study in 2 Belgian hospitals (September 2021-June 2023). Adults (aged ≥18 years) with MDD were identified via *DSM-IV* (*Diagnostic and Statistical Manual of Mental Disorders* [Fourth Edition]) and *ICD-10* (*International Statistical Classification of Diseases, Tenth Revision*) codes or natural language processing-detected note mentions; bipolar depression was excluded. TRD was defined as initiation of a third distinct antidepressant, supplemented by explicit mentions of TRD in notes. Structured data (demographics, diagnoses, medications, and hospitalizations) were harmonized in an Observational Medical Outcomes Partnership warehouse. Free-text notes were processed with a natural language processing pipeline to capture symptoms, psychiatric comorbidities, and contextual events.

**Results:**

We identified 1147 adults with MDD, of which 46% (524/1147) met TRD criteria. Females comprised 62.9% (722/1147) and mean (SD) age was 57.8 (18.4) years. Mortality was 13.3% (152/1147) overall (57/1147, 10.9% TRD vs 95/1147, 15.2% non-TRD). Common medical comorbidities were central nervous system diseases (477/1147, 41.6%) and heart diseases (349/1147, 30.4%). Dementia was more frequent in TRD (42/1147, 8% vs 32/1147, 5.1%), whereas obesity was higher in non-TRD (70/1147, 11.2% vs 46/1147, 8.8%). Anxiety disorder occurred in 35.4% (406/1147) overall and was more prevalent in TRD (229/1147, 43.7% vs 177/1147, 28.4%); personality and panic disorders also trended higher. Severity was sparsely documented (severe MDD 170/1147, 14.8%) and standardized scales were rarely recorded.

**Conclusions:**

We present a step-by-step artificial intelligence-supported methodology tailored for clinicians, discussing challenges in integrating RWE into psychiatry, and identifying opportunities to enhance data collection with minimal workflow changes, which emphasizes the transformative potential of RWE systems in mental health research.

## Introduction

Major depressive disorder (MDD) affects a considerable portion of the global population, often leading to significant disability [1]. Meanwhile, treatment-resistant depression (TRD), where patients do not respond adequately to at least 2 standard antidepressant treatments, remains an especially difficult clinical challenge [2]. Studies have shown that patients with TRD often face poorer outcomes, which amplifies the need for tailored therapeutic approaches [2]. Beyond treatment nonresponse, TRD is also characterized by substantial clinical heterogeneity (eg, comorbidity profiles, symptom expression, and illness course), which complicates comparisons across cohorts and health systems and makes baseline characterization clinically relevant in its own right [3]. Large-scale electronic health record (EHR)—based studies have previously used TRD as a model phenotype and highlighted the need for reliable approaches to define outcomes and longitudinal status in routine care [4]. The MOOD project focuses on a comprehensive description of this patient population, aiming to elucidate the demographics, medical history, and psychiatric patient history among MDD and TRD cohorts.

Real-world evidence (RWE) has become increasingly important to complement or expand upon findings from controlled trial settings and fill data gaps on new treatments, for example, patient profiling [5]. Furthermore, natural language processing (NLP) offers a time-efficient means to mine free-text fields, which are abundant in psychiatric reports [6]. This is particularly relevant in mental health EHRs because clinically decisive information such as symptom clusters, course specifiers, contextual triggers, and clinician-assessed status frequently appears in narrative documentation rather than in standardized structured fields [4,6,7]. Moreover, psychiatric research is hindered by a lack of standardized documentation, particularly for severity levels and outcomes, which are often inconsistently captured or absent in structured data [8-10]. Empirical work has also shown that even when severity indicators exist (eg, patient health questionnaires), their availability can be incomplete or uneven across encounters [11], reinforcing the need to combine structured fields with information from clinical text when feasible. This work showcases the methodology of a pilot project in psychiatry aimed at streamlining the comprehensive analysis of MDD and TRD patient populations.

We present a detailed methodology of a preliminary study conducted at 2 Belgian hospitals. The study leverages NLP as an artificial intelligence approach to analyze unstructured data alongside structured data from EHRs in psychiatry [12-15], generating RWE that seeks to offer a nuanced understanding of patient experiences and treatment trajectories even in the absence of depression scale scores. In doing so, the approach aligns with broader developments in mental health informatics, where NLP has been used to structure depression-related information from large EHR repositories to support observational research at scale [7]. Given the clinician-facing orientation of this manuscript, the NLP workflow is described step-by-step with emphasis on interpretability, validation, and how outputs map back to clinical concepts, consistent with clinician-oriented guidance on the development and implementation of AI methods in medicine [16]. Initial results on patient demographics and comorbidities are included here, and future analyses will address patterns of care. The ultimate goal is to inform clinical practice and guide future research into the complexities of treating depression in diverse patient cohorts.

The MOOD project comprises three overarching objectives focused on patients with MDD, with particular emphasis on those meeting criteria for TRD. The first objective is to characterize patient demographics, medical history, and psychiatric disease burden, including MDD severity, in these populations. The second objective is to assess real-world treatment patterns, encompassing pharmacological therapies, psychotherapy, and neuromodulation approaches used in MDD and TRD. The third objective aims to address data gaps for emerging treatments, with a specific focus on treatment patterns associated with esketamine nasal spray in patients with TRD [3]. While the MOOD project encompasses multiple objectives, the comprehensive real-world characterization of patients with MDD and treatment-resistant depression represents an important scientific objective in its own right. Despite the clinical relevance of TRD, contemporary data describing disease severity, comorbidity burden, and longitudinal clinical profiles in routine care remain limited [8-10]. The present manuscript addresses this gap by reporting the results of the first project objective, thereby establishing a baseline understanding of the studied population prior to analysis of treatment patterns and outcomes, which are ongoing.

## Methods

### Study Design and Data Sources

This study was a preliminary multicenter analysis conducted at 2 Belgian hospitals (University Hospital Brussels and Psychiatric Center Ariadne) from September 2021 to June 2023. Data were extracted from both structured and unstructured sources within the EHRs of the hospital. Structured data encompasses demographic details, treatment dosages, frequency of administration, and hospitalization information, whereas unstructured data comprised clinicians’ notes, pathology reports, and other textual documentation that captured patient histories and treatment responses.

**Figure 1:**
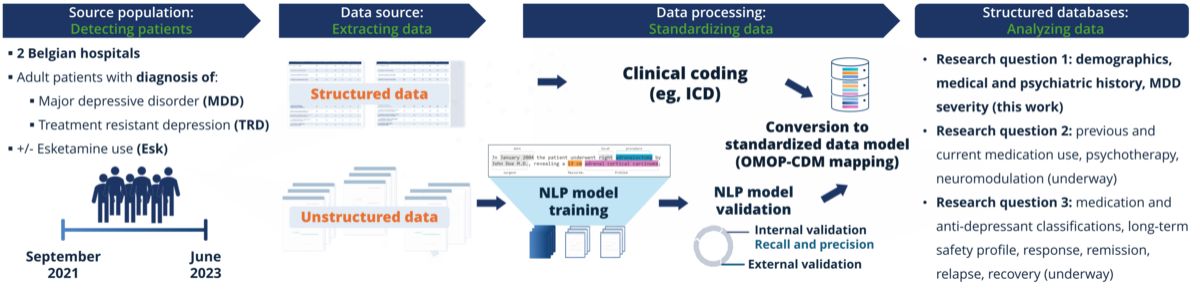
Schematic of the study design and methodology. Results for Research question one are included in this work, while analyses for Research questions 2 and 3 are underway. Esk: esketamine; *ICD*: *International Classification of Diseases*; MDD: major depressive disorder; NLP: natural language processing; OMOP-CDM: Observational Medical Outcomes Partnership-common data model; TRD: treatment resistant depression.

The integration of data sources facilitated a thorough analysis of patient characteristics and enabled the study to capture information frequently overlooked in traditional research settings, such as nuanced clinical observations noted only in free-text fields. As the available data originate from two Belgian hospitals, the findings reflect the clinical documentation structure of these settings, with potential for broader generalizability as additional sites are incorporated in future work. A schematic of the overall study design is provided in Figure 1.

### Participants and Cohorts

Patients eligible for inclusion were those aged 18 years or older with any record of MDD or TRD, and some had documented esketamine (Esk) use. Treatment‑resistant depression is defined by the European Medicines Agency (EMA) as MDD with inadequate response to at least 2 different antidepressant treatments administered at adequate dose and duration [17]. Likewise, EMA approved esketamine specifically for adults with TRD [18], such that patients with TRD were assumed to have a diagnosis of MDD, and patients with esketamine use were assumed to have both MDD and TRD. This operational definition prioritizes sensitive real-world identification of TRD in EHR data; reasons for treatment changes (eg, nonresponse vs intolerance) are not consistently documented in structured records and could not be reliably distinguished. This definition is also consistent with prior large-scale TRD studies such as STAR*D [19], which similarly rely on sequencing of antidepressant trials in the absence of systematically documented reasons for change [20-22].

**Figure 2:**
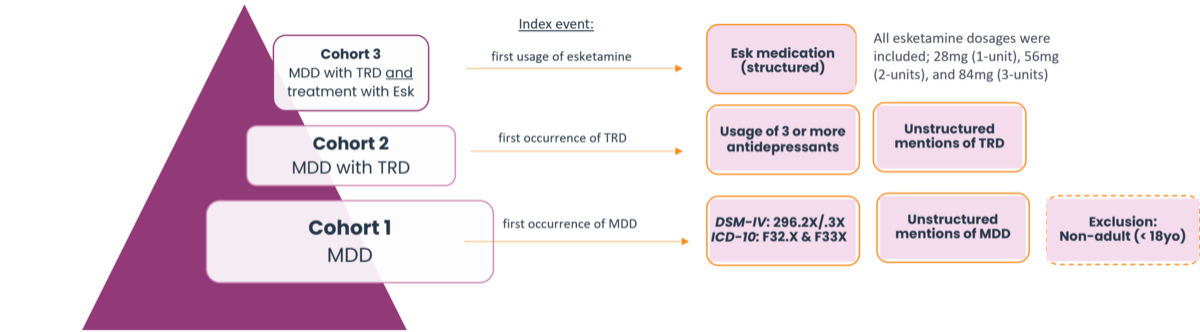
Schematic of the nested cohorts included in the study, their index events, and definitions. *DSM-IV: Diagnostic and Statistical Manual of Mental Disorders, Fourth Edition*; Esk: esketamine; *ICD-10*: *International Classification of Diseases*, Tenth Revision; MDD: major depressive disorder; TRD: treatment resistant depression.

Three nested cohorts were established (Figure 2), including a MDD cohort (diagnosed through *ICD-10* [*International Statistical Classification of Diseases, Tenth Revision*] codes F32.X or F33.X, as well as *DSM-IV* [*Diagnostic and Statistical Manual of Mental Disorders* {Fourth Edition}] and *DSM-5* [*Diagnostic and Statistical Manual of Mental Disorders* {Fifth Edition}] codes 296.2X, 296.3X, excluding bipolar depression), a TRD cohort (individuals with three or more distinct antidepressants during the study period, or explicit mention of TRD in unstructured notes), and an esketamine (Esk) cohort (patients with esketamine treatment events of any dose). The index date for the MDD cohort was defined as the first occurrence of either MDD, TRD, or Esk, for the TRD cohort as the first occurrence of either TRD or Esk, and for the Esk cohort as the first recorded esketamine administration. Maximum follow-up (censoring) was applied based on the latest recorded date for any event per patient, defined as any recorded date for medication, diagnosis, or treatment in both structured and unstructured data.

### Variables

The variables were grouped according to the MOOD study’s primary objectives, with corresponding timeframes and patient cohorts of interest (Table 1). This study presents the results for demographics, medical history, and psychiatric history, which constitute the first objective (shown as “this work” in Table 1). Future analyses will cover the additional variables linked to pharmacological and psychotherapeutic interventions, esketamine treatment patterns, and long-term outcomes (shown as “ongoing” in Table 1). Specific medications and antidepressant classifications can be found in Table S1 in Multimedia Appendix 1.

**Table 1:** Variables used in the study per objective, with their respective cohorts and timeframe.

Objective	Variables extracted	Cohorts	Timeframe
Patient demographics, medical, and psychiatric history (this work)	Demographics: age, sex, death status. Medical history: Parkinson disease, obesity, cardiac arrhythmia, rheumatoid arthritis, kidney disease, COPD^a^, stomach disease, heart disease, anemia, central nervous system diseases, dementia, liver disease, HIV, diabetes, thyroid disease, cancer, stroke, CVS^b^ or fibromyalgia, hypercholesterolemia, autoimmune disease, infectious diseases, and hypertension. Psychiatric history: borderline personality disorder, bulimia nervosa, obsessive-compulsive disorder, circadian rhythm disorders, anxiety disorder, addiction (composite), personality disorder, panic disorder, posttraumatic stress disorder, anorexia nervosa, suicidal tendencies. MDD^c^ severity: mild, moderate, and severe MDD, MADRS^d^, CGI-S^e^.	MDD and TRD^f^ (this work), Esk^g^ (ongoing)	On or before index date
Medication, psychotherapy, and neuromodulation treatments (ongoing)	Medication use: SSRIs^h^, tricyclic antidepressants, atypical antipsychotic augmentation, serotonin and noradrenaline reuptake inhibitors, monoamine oxidase inhibitors, anxiolytics, selective noradrenaline reuptake inhibitors, antidepressants acting on neuroreceptors, noradrenaline and dopamine reuptake inhibitors, other antidepressants. Psychotherapy: CBASP^i^, cognitive behavioral therapy, psychoanalytic therapy, crisis intervention, psychodynamic psychotherapy, problem-solving therapy, mindfulness, relationship or family therapy, interpersonal psychotherapy, general psychotherapy. Neuromodulation: transcranial magnetic stimulation and electroconvulsive therapy.	TRD and Esk	On or before index date
Esketamine treatment patterns (ongoing)	Treatment characteristics: Frequency, duration, dosing, switching, and cessation. Efficacy: Response, remission, relapse, recovery. Safety: Adverse events. Psychiatric hospitalization.	Esk	Within esketamine treatment period

^a^COPD: chronic obstructive pulmonary disorder.

^b^CVS: cyclic vomiting syndrome.

^c^MDD: major depressive disorder.

^d^MADRS: Montgomery-Åsberg Depression Rating Scale.

^e^CGI-S: Clinical Global Impression - Severity Scale.

^f^TRD: treatment-resistant depression.

^g^Esk: esketamine.

^h^SSRI: selective serotonin reuptake inhibitor.

^i^CBASP: cognitive behavioral analysis system of psychotherapy.

### Database Generation

Following data extraction, the processed information was systematically integrated into a research-grade Observational Medical Outcomes Partnership (OMOP) Common Data Model (CDM) data warehouse, which promotes consistent data storage and reproducibility [23]. Data mapping used the *ICD* (*International Classification of Diseases*) codes for structured data and their equivalent Unified Medical Language System (UMLS) Concept Unique Identifiers (CUIs), while unstructured data were directly encoded to CUIs (see full data point list in Table S2 in Multimedia Appendix 1).

NLP pipeline as an initial step, the underlying clinical notes were characterized by determining the total number of records, the number of patients with at least one record, and the allocation of documents to the predefined note‑type categories. These data informed the preprocessing stages of the NLP pipeline.

NLP was used to handle unstructured text derived from clinical notes, pathology reports, and other sources. In this pipeline, text preprocessing involved splitting documents into sentences and words, followed by entity recognition to identify clinical terms such as “remission” or “relapse.” Entity linking was performed to map recognized entities to standardized medical concepts in the UMLS. An attribute extraction phase was then used to capture further details such as negation (eg, “no suicidal ideation”) or speculation. Model training and fine-tuning leveraged extensive medical datasets, with adjustments made for the psychiatry context, as shown for other therapeutic areas in detail (see [12-15,24,25]).

**Figure 3:**
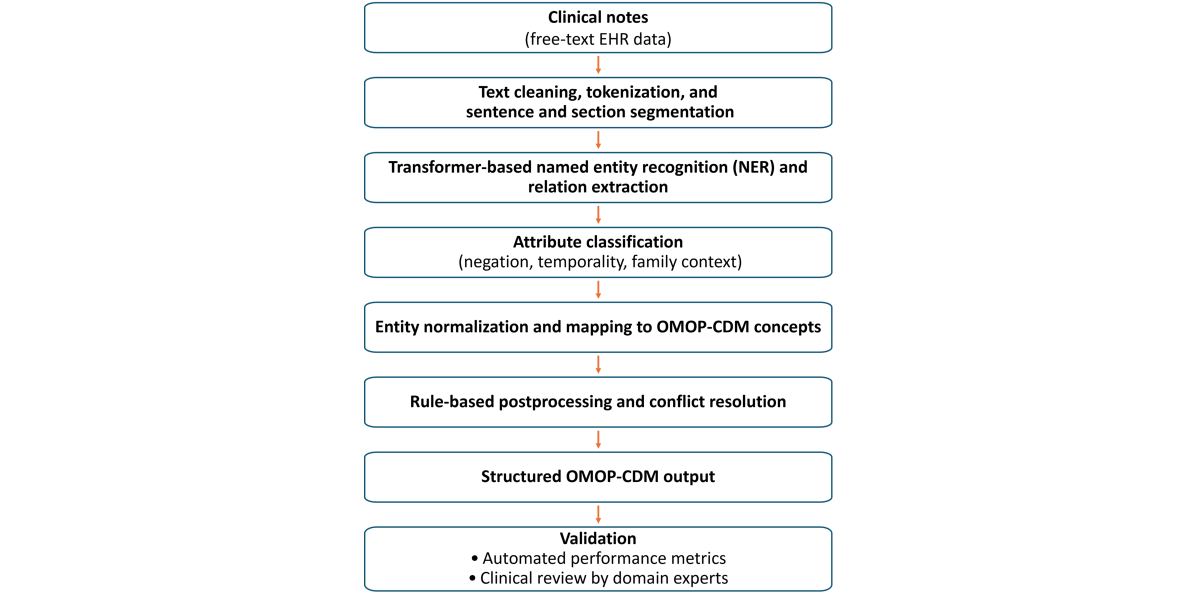
Overview of the clinical natural language processing pipeline used to convert free-text electronic health record documentation into structured Observational Medical Outcomes Partnership-common data model outputs. The workflow begins with clinical notes and applies text cleaning and segmentation, transformer-based named entity recognition and relation extraction, and attribute classification (eg, negation, temporality, and family context). Extracted mentions are then normalized to standardized Observational Medical Outcomes Partnership-common data model concepts, followed by rule-based post-processing and conflict resolution. The resulting structured Observational Medical Outcomes Partnership-common data model dataset is evaluated using automated performance metrics and clinical review by domain experts. EHR: electronic health record; NER: named entity recognition; OMOP-CDM: Observational Medical Outcomes Partnership-common data model.

Figure 3 provides an overview of the end-to-end workflow, from free-text EHR documentation to structured, analysis-ready OMOP-CDM outputs, and situates each NLP component within the overall data-generation process. In line with the accepted methodological description of our platform and prior validated implementations in other disease areas [12-15,24-26], the pipeline was implemented as a cascade of transformer-based models complemented by rule-based interventions defined by domain experts to support robust extraction in real-world clinical text.

Text cleaning, tokenization, and sentence and section segmentation were first applied to standardize heterogeneous document formats and to support downstream extraction within meaningful clinical contexts (Figure 3). Transformer-based named entity recognition (NER) and relation extraction were then applied to identify clinically relevant entities (eg, symptoms, diagnoses, and medications) and their relations (eg, date or dosage). In the pipeline, NER and relation extraction are framed as a sequence-to-sequence structured prediction task, which generates a structured representation of entities and relations directly from raw text [25,26]. This approach supports joint extraction of entities and relations in a single step and allows nested or compositional mentions to be represented in the model output.

After entity extraction, attribute classification was used to capture the contextual status of each extracted mention, including negation, temporality, and family context (Figure 3). This step distinguishes affirmed findings from negated or uncertain statements (eg, “no suicidal ideation,” “possible relapse,” “history of depression,” or “family history of bipolar disorder”), thereby reducing false positives that can arise when narrative documentation contains ruled-out symptoms or historical information. The attribute model is fine-tuned for multiple Boolean or contextual attributes, enabling classification of mentions into categories such as absent or negated, hypothetical, historical, or family-related [25,26].

Entity normalization and mapping to standardized OMOP-CDM concepts were subsequently performed (Figure 3). Structured data elements retained their source coding (eg, *ICD* where applicable) and were aligned to standardized concept identifiers, while extracted unstructured mentions were normalized to UMLS-linked concepts and mapped to the OMOP-CDM vocabulary. Normalization is implemented via transformer-based entity linking (eg, SapBERT-style metric learning over UMLS) with nearest-neighbor retrieval and threshold optimization; concept mapping covers relevant clinical vocabularies used within OMOP (eg, SNOMED CT [Systematized Nomenclature of Medicine – Clinical Terms], *ICD-10*, Logical Observation Identifiers Names and Codes [LOINC], and RxNorm) [25,26].

Finally, rule-based postprocessing and conflict resolution were applied to improve data fidelity and address recurrent real-world documentation artifacts (Figure 3). These interventions include pattern-based extraction for semistructured mentions, exclusion of nonclinical document sections, filtering of incompatible NER label-semantic type combinations, resolution of conflicting attributes (eg, historical labels paired with inconsistent dates), and default date assignment when timing is not explicitly documented, using a predefined cascade of rules [25,26].

### Training Data and Annotation Framework

The NLP models, used for initial processing of the corpus, rely on a continuously expanding proprietary corpus of manually annotated clinical text in Dutch. This annotated resource includes approximately 33897 sentence‑level annotations across 5954 sentences (for NER, relation extraction, and attribute detection) and 4716 concept‑level annotations from 3777 documents (for coding and attribute classification). For this study, model performance was estimated using a separate set of 2638 clinician‑validated annotations derived from 1921 documents, which serve as an out‑of‑the‑box (OOTB) evaluation sample. These validation annotations were subsequently reintegrated into the training pool as part of an iterative continuous‑learning process, in which corrected model outputs inform further fine‑tuning. This workflow reflects our standard development approach, prioritizing broad clinical coverage and iterative refinement rather than fixed, project‑specific data partitions.

### Validation

Validation involved a multistep process to confirm data quality and clinical reliability (Figure 3). First, automated evaluation was performed by comparing NLP-extracted concepts against a clinician-curated gold standard, with precision, recall, and *F*_1_ scores calculated at the datapoint level; where relevant, performance was also assessed separately for specific subtasks such as attribute handling (eg, negation and temporality) and concept normalization. Second, clinical review by domain experts was conducted on sampled patient records and on aggregated outputs to confirm that extracted concepts were clinically meaningful, internally consistent, and aligned with expectations from routine practice. This review step explicitly targeted common failure modes in psychiatric documentation, including ambiguous phrasing, historical versus current symptom mentions, and “rule-out” statements, and it served as an iterative quality check to identify systematic extraction errors that could affect downstream analyses. Findings from automated metrics and expert review informed targeted refinements of extraction logic and postprocessing (eg, resolving conflicting attributes or excluding nonclinical document sections), followed by reassessment to ensure that updates improved fidelity without introducing new inconsistencies [25,26].

### Statistical Analysis

Descriptive statistics were used to summarize findings related to the first objective (patient demographics, medical history, and psychiatric history). Means, medians, SDs, and minimum-maximum values were used for continuous variables such as age at index, whereas categorical variables, including comorbidity status, were presented as frequencies and percentages. Statistical analyses were performed using R environment software (version 4.1.0; R Foundation for Statistical Computing). No inferential analyses were included because the objective was descriptive cohort characterization rather than hypothesis testing. Counts below five were suppressed as “<5” (<x%), and when this alone still allowed back-calculation, the complementary subgroup was shown as a symmetric count and percentage range.

### Ethical Considerations

Ethical approval was obtained from the relevant Institutional Review Boards (Commissie Medische Ethiek, Universitair Ziekenhuis Brussel, BUN1432023000167, EC-2023-199, Project ID=23162_RWE Mood). The ethics evaluation resulted in approval for the secondary use of routinely collected health data. The study was conducted following the Declaration of Helsinki, ethical standards of the responsible institutional and national committees on human experimentation, and applicable regulations. Patient privacy was safeguarded according to Belgian Privacy Law and General Data Protection Regulation articles 6, 9, and 89. Data were pseudonymized prior to analysis. Patient consent was waived by the approving ethics committees due to pseudonymization of the data and the retrospective nature of the study, in accordance with Belgian legislation. No financial or other compensation was provided to patients.

## Results

### NLP Pipeline and Metrics

A total of 56,396 records across both hospitals were included in the dataset, where 1262 patients presented at least one record. Clinical assessment notes constituted the majority of all extracted records (32,075/56,396, 56.9%). Admission, intake, and hospitalization notes (13,109/56,396, 23.2%), together with consultation notes (5605/56,396, 9.9%, including client, referrer, bedside, psychiatric, and telephone contacts), accounted for most of the remaining documentation. The other note types, including multidisciplinary meeting reports, administrative or system‑generated communications, diagnostic or procedural documents, risk and safety assessments, and treatment‑plan notes, each represented a smaller proportion of the dataset (< 4% each).

Performance against a clinician-annotated gold standard, reported as precision and recall for OOTB pipeline versus the postmitigation pipeline (ie, after rule-based postprocessing and conflict resolution), is summarized in Table S3 in Multimedia Appendix 1. Overall, postmitigation precision was high, with values ranging from 66.7% to 100% (96.6% mean), while postmitigation recall ranged from 91.7% to 100% (98.1% mean). In contrast, OOTB recall was notably more variable (range 10%-100%, 72.3% mean), supporting the importance of the mitigation layer for reliable extraction.

Performance gains were most visible for clinically central symptom and outcome concepts that are frequently documented in narrative form. For example, recall improved from 39.3% to 95.2% for depressed mood, from 42.4% to 93.9% for anxiety, and from 35.4% to 95.8% for suicidal mentions. Similarly, the concept Major Depressive Disorder increased from 10% to 95% recall after mitigation, indicating that postprocessing materially reduced missed detections for key diagnostic constructs when expressed variably in free text (Table S3 in Multimedia Appendix 1).

Across variable classes, medication entities and medication-class groupings tended to perform best after mitigation (typically near-perfect precision and recall for commonly observed treatments or classes in the annotated sample), as did many diagnosis or comorbidity concepts (postmitigation precision typically in the high-90% range with recall approaching 100%). Symptom or context concepts also reached strong postmitigation recall overall, but they represented the main area where OOTB performance was lower, consistent with the greater linguistic variability of symptom documentation in psychiatric notes.

Routine documentation sometimes indicated different intended uses for the same medication, which is not consistently recoverable from structured medication records alone. For example, trazodone and benzodiazepines were frequently mentioned in the context of sleep rather than antidepressant or anxiolytic use, and this indication context was captured from unstructured notes by the NLP extraction layer.

### Patient Demographics

Table 2 summarizes key demographic characteristics of patients with MDD, stratified into not-treatment-resistant (Not TRD, 623/1,147, 54%) and treatment-resistant (TRD, 524/1,147, 46%) subgroups. Among patients with TRD, 2% (n=8) had an explicit TRD mention in clinical notes; the remaining patients met the TRD definition based on medication information (≥3 distinct antidepressants). Females comprise 62.9% (722/1,147) of the overall MDD population, with a higher proportion in the TRD group (349/524, 66.6%) compared to Not TRD (373/623, 59.9%). The mean age is 57.8 years, and the median age is slightly higher in the TRD group (60 years) than in not TRD (58 years). Most patients are alive (995/1,147, 86.7%), with a slightly higher mortality rate in the Not TRD group (95/623, 15.2%) compared to TRD (57/524, 10.9%).

**Table 2:** Patient demographics.

Characteristics			MDD^a^ (Overall), n (%)		MDD (N=1147; 100%)		
					Not TRD^b^, n (%) (N=623; 54% of MDD)		TRD, n (%) (N=524; 46% of MDD)
**Sex, n (%)**							
	Female	722 (62.90)		373 (59.90)		349 (66.60)	
	Male	425 (37.10)		250 (40.10)		175 (33.40)	
**Age at index**							
	Mean (SD)	57.8 (18.4)		57.2 (18.7)		58.4 (18)	
	Median (min-max)	59 (18-101)		58 (19-96)		60 (18-101)	
**Status, n (%)**							
	Alive	995 (86.70)		528 (84.80)		467 (89.10)	
	Dead	152 (13.30)		95 (15.20)		57 (10.90)	

^a^MDD: major depressive disorder.

^b^TRD: treatment-resistant depression.

### Medical and Psychiatric History of MDD and Patients With TRD

Table 3 summarizes the medical and psychiatric history of patients with MDD, with or without TRD. Among medical comorbidities, central nervous system diseases, infections, and heart diseases are the most common, with similar prevalence across both subgroups. Notable differences include higher rates of dementia in patients with TRD (42/524, 8% vs 32/623, 5.1%) and obesity in individuals without TRD (70/623, 11.2% vs 46/524, 8.8%). Psychiatric comorbidities reveal more pronounced differences, with anxiety disorders found at higher proportions for patients with TRD (229/524, 43.7% vs 177/623, 28.4%), along with higher rates of personality disorders and panic disorders. Alcohol and drug-related issues are prevalent in both groups, with slightly higher alcohol abuse (148/623, 23.8%) and drug dependence (152/623, 24.4%) in patients without TRD. In terms of depression severity, detected on or at the index date, severe MDD is observed in 14.8% (170/1147) of the overall population, with comparable rates across subgroups. Severity was only well captured in a minority of patients (see the Discussion section). This data underscores the complex clinical profiles of MDD and highlights key differences between treatment-resistant and nontreatment-resistant cases.

**Table 3:** Patient history.

Characteristics		MDD^a^ (Overall) (N=1147)	MDD (N=1147; 100%)	
			Not TRD^b^ (N=623; 54% of MDD)	TRD (N=524; 46% of MDD)
**Medical history, n (%)**				
	Anemia	175 (15.3)	94 (15.1)	81 (15.5)
	Autoimmune disease	33 (2.9)	18 (2.9)	15 (2.9)
	Cancer	116 (10.1)	58 (9.3)	58 (11.1)
	Cardiac arrhythmia	210 (18.3)	112 (18)	98 (18.7)
	Central nervous system diseases	477 (41.6)	261 (41.9)	216 (41.2)
	COPD^c^	100 (8.7)	59 (9.5)	41 (7.8)
	CVS^d^ or fibromyalgia	38 (3.3)	10 (1.6)	28 (5.3)
	Dementia	74 (6.5)	32 (5.1)	42 (8)
	Diabetes	193 (16.8)	121 (19.4)	72 (13.7)
	Heart diseases	349 (30.4)	190 (30.5)	159 (30.3)
	HIV	11 (1)	5 (0.8)	6 (1.1)
	Hypercholesterolemia	196 (17.1)	110 (17.7)	86 (16.4)
	Hypertension	306 (26.7)	169 (27.1)	137 (26.1)
	Infection	344 (30)	187 (30)	157 (30)
	Kidney disease	181 (15.8)	100 (16.1)	81 (15.5)
	Liver disease	107 (9.3)	60 (9.6)	47 (9)
	Obesity	116 (10.1)	70 (11.2)	46 (8.8)
	Parkinson	23 (2)	10 (1.6)	13 (2.5)
	Rheumatoid arthritis	6 (0.5)	<5^e^	<5
	Stomach disease	134 (11.7)	65 (10.4)	69 (13.2)
	Stroke or cerebrovascular accident	32 (2.8)	15 (2.4)	17 (3.2)
	Thyroid disease	153 (13.3)	78 (12.5)	75 (14.3)
**Psychiatric history, n (%)**				
	Alcohol abuse	250 (21.8)	148 (23.8)	102 (19.5)
	Anorexia nervosa	12 (1)	5 (0.8)	7 (1.3)
	Anxiety disorder	406 (35.4)	177 (28.4)	229 (43.7)
	Bipolar disorder	67 (5.8)	30 (4.8)	37 (7)
	Borderline personality disorder	43 (3.7)	19 (3)	24 (4.6)
	Bulimia nervosa	<5	<5	<5
	Circadian rhythm disorders	<5	<5	<5
	Drug abuse	85 (7.4)	37 (5.9)	48 (9.2)
	Drug dependence	254 (22.1)	152 (24.4)	102 (19.5)
	Obsessive-compulsive disorder	<5	<5	<5
	Panic disorder	13 (1.1)	<5	7-13 (1.3-2.5)
	Personality disorder	108 (9.4)	44 (7.1)	64 (12.2)
	Posttraumatic stress disorder	25 (2.2)	13 (2.1)	12 (2.3)
	Suicidal	334 (29.1)	174 (27.9)	160 (30.5)
	Addiction	298 (26)	165 (26.5)	133 (25.4)
	CGI-S^f^	—^g^	—	—
	MADRS^h^	—	—	—
	Mild major depressive disorder	<5	<5	<5
	Moderate major depressive disorder	22 (1.9)	15-21 (2.4-3.4)	<5
	Severe major depressive disorder	170 (14.8)	96 (15.4)	74 (14.1)

^a^MDD: major depressive disorder.

^b^TRD: treatment-resistant depression.

^c^COPD: Chronic Obstructive Pulmonary Disease.

^d^CVS: Cyclic Vomiting Syndrome.

^e^These groups contains less than 5 patients; hence, percentages are not available for these groups. Precise numbers are masked per the contract between the hospitals and the sponsor.

^f^CGI-S: Clinical Global Impressions-Severity.

^g^Not available.

^h^MADRS: Montgomery-Åsberg Depression Rating Scale.

## Discussion

### Overview, Scope, and Importance of the MOOD Project

The MOOD project highlights the growing need for RWE in psychiatry, particularly for understanding complex disorders such as MDD and its treatment-resistant form [8,27]. The findings presented here provide an essential real‑world baseline of disease burden and patient complexity in MDD and TRD, enabling more informed interpretation of subsequent treatment‑focused analyses from this project. By integrating structured and unstructured data, the study addresses key gaps in psychiatric research, where conventional methods often overlook contextual details captured only in free-text notes [12,13]. This approach is vital given the global prevalence and significant disease burden of depression, as well as the unique clinical and economic challenges posed by TRD [2,3]. Building upon existing literature that highlights the limitations of traditional datasets, the MOOD project aims to generate comprehensive insights that can inform a more patient-centered and evidence-driven model of psychiatric care [5,6,8].

### Main Findings and Link to Previous Work

Results regarding NLP metrics support the contribution of unstructured text augmentation: variables that are often absent or inconsistently represented in structured fields (symptoms, clinical course descriptors, response language) can be extracted with high postmitigation performance, aligning with prior evidence that NLP improves ascertainment beyond structured claims data alone in large-scale psychiatric EHR studies [4]. The first analyses from the MOOD project reveal how NLP-based extraction of free-text data complements structured clinical fields, leading to a richer depiction of patient trajectories in MDD and TRD. The systematization of clinical experience results in a holistic overview that avoids confirmation bias based on individual cases. [6,7]. For instance, the project’s NLP pipeline successfully extracted a dashboard overview of clinical experience in TRD.

Automation of data extraction through NLP further reduces the burden on clinicians, allowing them to spend more time with patients while still generating valuable research insights [6]. This aligns with the broader goal of enhancing both research and clinical practice in psychiatry. These findings align with prior large-scale investigations showing that real-world data capture a heterogeneous array of patient experiences and outcomes [8,27]. The capacity to identify these factors using textual data sources resonates with studies showing that TRD can have varying definitions and multistep strategies commonly are recommended, which may involve switching, augmentation, or combination regimens [3]. Consequently, the MOOD findings expand on this literature by illustrating how robust informatics frameworks can overcome the inconsistent or complex documentation required for psychiatric research.

As extensively discussed in major TRD reviews and large real-world cohorts [20-22], heterogeneity in TRD definitions and reliance on retrospective EHR documentation contribute to variability in reported prevalence; our findings therefore align with expectations for hospital-based MDD populations [22], where TRD proportions typically approach 45%-50% rather than the approximately 30% seen in general MDD populations [3]. In addition, the descriptive differences observed in our cohort, such as higher rates of dementia, anxiety disorders, personality disorders, and panic disorder among patients with TRD, are broadly consistent with patterns described in published European TRD cohorts, which similarly report greater psychiatric and medical complexity in TRD than non-TRD populations [28-30].

### Disentangling the Depression Symptom Cluster Using Unstructured Data

**Figure 4:**
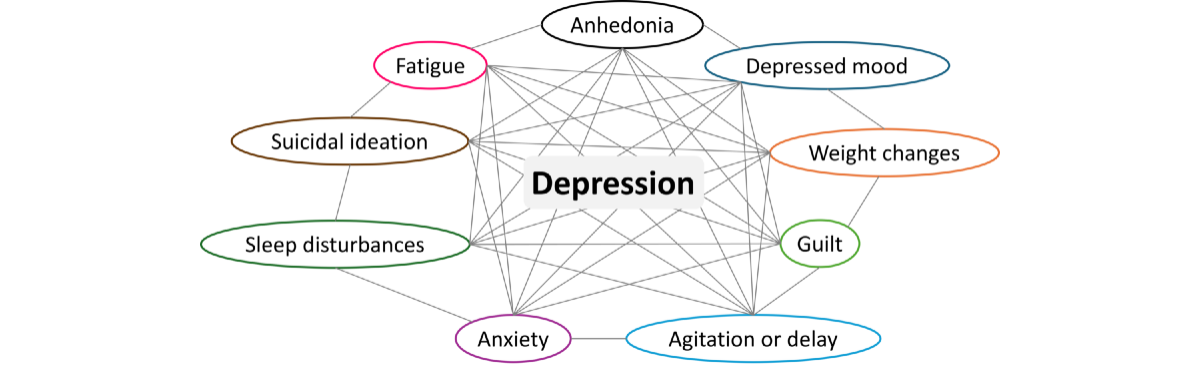
Schematic representing a network of the “nine pillars of depression”. Each node represents a key symptom or domain—such as anhedonia, depressed mood, weight changes, and sleep disturbances—which collectively define depression as a clinical syndrome. The network schematic emphasizes how these interconnected elements comprise the overarching concept of depression [26].

The study highlights the importance of a structured framework for understanding the “nine pillars of depression,” or “depression symptom cluster” that defines MDD in the *DSM-V* [31], which encompasses key domains such as diagnosis, symptom severity, and quality of life (Figure 4). While structured data sources offered a baseline for documenting diagnoses and comorbidities, unstructured data provided critical insights into the nuanced clinical profiles of patients. Indeed, patient profiles within MDD can vary widely depending on symptom patterns [32]. Through the use of the NLP pipeline, descriptions of symptom clusters, such as anhedonia, psychomotor retardation, and psychosocial stressors, can be more easily extracted, offering a more comprehensive understanding of patient experiences. This approach addresses the challenges inherent in psychiatric data, where nonstandardized language can obscure critical details about each patient’s depression severity or clinical features. By extracting and categorizing these domains via NLP, researchers gain a more nuanced view of how depression manifests and evolves in real-world settings, supporting calls for thorough documentation in clinical practice.

The depression symptom cluster includes the following symptom domains:

1. Anhedonia
2. Depressed mood
3. Changes in weight or appetite
4. Sleep disturbances
5. Psychomotor agitation or retardation
6. Feelings of anxiety
7. Feelings of guilt or worthlessness
8. Fatigue or diminished concentration
9. Suicidal ideation or thoughts of self-harm

### Challenges in Real-World Psychiatric Research

Despite its advantages, leveraging RWE in psychiatry presents notable challenges. A primary issue is the inconsistent documentation of critical clinical parameters [6]. Depression severity, for example, is often recorded in free-text notes without the use of standardized scales such as the Montgomery-Åsberg Depression Rating Scale (MADRS) or the Clinical Global Impression - Severity (CGI-S) scale [8]. This lack of standardization complicates the extraction and analysis of severity data [9,10]. With minimum extra work on behalf of the physicians, that is, noting down CGI-S scores, the clinical added value to be gained from patient records would be notable.

Unstructured clinical notes, while rich in contextual information, pose additional difficulties for data extraction. NLP tools must contend with variability in language, medical jargon, and incomplete or ambiguous entries. Recent advancements in NLP have improved the accuracy of concept extraction, yet challenges remain, particularly in capturing nuanced information such as reasons for treatment changes or patient-reported outcomes [6,33].

The integration of structured and unstructured data also requires careful attention to data quality and consistency. Discrepancies between these sources can lead to errors in analysis or misinterpretation of findings. For example, structured data may indicate a medication switch, but unstructured notes may reveal that the switch was due to adverse effects rather than lack of efficacy [6,8,27].

Our findings also revealed significant variability in the documentation and conceptualization of TRD [3]. We defined TRD as the initiation of a third antidepressant during the study period, as identified through structured data sources. However, unstructured notes frequently included additional nuances, such as mentions of partial response, adverse effects, or patient-reported discontinuation reasons. While the EMA’s definition of TRD relies on two failed antidepressants in one same depressive episode [3], medication failure (eg, due to side effects or inefficacy) was not reliably captured in structured data alone. NLP contributed by identifying unstructured mentions of TRD that provided context for treatment changes, supporting the ongoing refinement of TRD criteria for clinical use.

### Limitations of the MOOD Project

While the MOOD project represents a significant step forward, several limitations must be acknowledged. First, the study relied on data from two hospitals in Belgium, so future expansions to include additional sites and more diverse datasets could broaden the extent of the findings. Second, the accuracy of the NLP pipeline, while robust, is contingent on the quality and completeness of the underlying data. Missing or ambiguous information in clinical notes can affect the reliability of extracted concepts, and variability in clinician terminology limits the accuracy of NLP tools, a challenge noted across studies leveraging NLP for mental health interventions [6]. For instance, this analysis did not attempt to algorithmically infer or predict depression severity scores when not documented; severity capture was descriptive and limited to explicitly recorded severity descriptors and scale measurements identified in structured fields or narrative notes. Efforts to standardize documentation practices and improve data curation are critical for addressing these issues [9]. Finally, the lack of patient-reported outcomes in the study highlights a gap in understanding the subjective experiences of patients with MDD and TRD. Incorporating patient-reported measures in future studies could provide a more comprehensive view of treatment efficacy and quality of life [33].

### Conclusions

The findings from the MOOD project underscore the importance of continued innovation in RWE methodologies for psychiatry. Expanding the study to additional hospitals and incorporating standardized documentation practices will enhance the generalizability of the results. RWE studies also have the potential to improve clinical decision-making. By uncovering patterns in real-world treatment use, such as the adoption of esketamine for TRD, these studies can guide the development of more effective and personalized care strategies [8,34,35]. Moreover, the ability to analyze patient trajectories in diverse populations can help address disparities in access to and outcomes of psychiatric care [9,34]. Furthermore, integrating patient-reported outcomes and exploring the use of novel data sources, such as wearable devices or mobile health applications, could enrich future research [33,36]. Collaboration between clinicians, data scientists, and policymakers is essential to ensure that RWE studies address the most pressing challenges in psychiatric care. By leveraging advancements in artificial intelligence and NLP, future projects can build on the foundation laid by the MOOD project, ultimately improving outcomes for patients with MDD, TRD, and other mental health conditions. Therefore, the MOOD project can be a first cornerstone for cross-border collaboration on a structured RWE network for mental health.

## Appendix

Multimedia Appendix 1 Additional tables.

## Abbreviations

CDM: common data model
CGI-S: clinical global impressions-severity
CUI: concept unique identifier
DSM-IV: Diagnostic and Statistical Manual of Mental Disorders (Fourth Edition)
DSM-V: Diagnostic and Statistical Manual of Mental Disorders (Fifth Edition)
EHR: electronic health record
EMA: European Medicines Agency
ICD: International Classification of Diseases
ICD-10: International Statistical Classification of Diseases, Tenth Revision
MADRS: Montgomery-Åsberg Depression Rating Scale
MDD: major depressive disorder
NLP: natural language processing
OMOP: Observational Medical Outcomes Partnership
NER: named entity recognition
OOTB: out‑of‑the‑box
RWE: real-world evidence
SNOMED CT: Systematized Nomenclature of Medicine – Clinical Terms
TRD: treatment-resistant depression
UMLS: Unified Medical Language System

## Data Availability

The datasets generated or analyzed during this study are not publicly available due to them being property of each participating hospital and therefore subject to strict privacy laws, but are available from the corresponding author on reasonable request.
