# Supplementary material for: Real‑World Clinical Characterization of Major Depressive Disorder and Treatment‑Resistant Depression Supported by Natural Language Processing: Multicenter Observational Study From the MOOD Project
Source: Interact J Med Res. 2026 May 22;15:e86448. doi: 10.2196/86448 (PMC13241795; doi:10.2196/86448)
Supplement: Multimedia Appendix 1 [file ijmr_v15i1e86448_app1.docx]

**Supplementary information**

**Supplementary Table 1.** Medication and antidepressant classification

| **Classification** | **Medication** | **Antidepressant** |
| --- | --- | --- |
| **Selective serotonin reuptake inhibitors (SSRIs)** | Citalopram, Escitalopram, Fluoxetine, Fluvoxamine, Paroxetine, Sertraline | Yes |
| **Serotonin and noradrenaline reuptake inhibitors** | Duloxetine, Venlafaxine | Yes |
| **Selective noradrenaline reuptake inhibitors** | Reboxetine | Yes |
| **Noradrenaline and dopamine reuptake inhibitors** | Bupropion | Yes |
| **Tricyclic antidepressants** | Amitriptyline, Clomipramine, Dosulepine, Imipramine, Nortriptyline | Yes |
| **Monoamine oxidase inhibitors** | Moclobemide | Yes |
| **Antidepressants acting on neuroreceptors** | Agomelatine, Mianserine, Mirtazapine, Trazodone | Yes |
| **Antidepressants (other)** | Esketamine, Vortioxetine | Yes |
| **Augmentation - atypical antipsychotics** | Quetiapine, Aripiprazole, Risperidone, Olanzapine, Lithium | No |
| **Anxiolytics** | Lorazepam, Alprazolam, Clonazepam, Diazepam, Zolpidem, Zopiclone | No |
| **Drugs used for bipolar disorder**  **Or as Augmentation** | Lithium, Valproate, Quetiapine, Lamotrigine, Carbamazepine | No |

**Supplementary Table 2.** Full data point list

| **Datapoint Name** | **Source** | **OMOP table** | **UMLS CUI Descendants** |
| --- | --- | --- | --- |
| Adverse reaction to antidepressant | All | Condition occurrence | C0569546, C0569556, C0569542 |
| Agomelatine | All | Drug exposure | C0971637 |
| AIDS | All | Condition occurrence | C0001175 |
| Alcohol abuse | All | Observation | C0085762 |
| Allergic Reaction | All | Condition occurrence | C1527304 |
| Amisulpride | All | Drug exposure | C0103045 |
| Amitriptyline | All | Drug exposure | C0002600 |
| Anemia | All | Condition occurrence | C0002871 |
| Anhedonia | Unstructured | Condition occurrence | C0178417 |
| Anorexia nervosa | All | Condition occurrence | C0003125 |
| Antidepressant therapy | All | Procedure occurrence | C1096649 |
| Antidepressants | All | Drug exposure | C0003289 |
| Antidepressants (other) | All | Drug exposure | C0813171, C2825616, C3661282 |
| Antidepressants acting on neuroreceptors | All | Drug exposure | C0025912, C0971637, C0040805, C0049506 |
| Antipsychotic drug therapy | All | Procedure occurrence | C1319421 |
| Antipsychotics | All | Drug exposure | C0040615 |
| Anxiety | Unstructured | Condition occurrence | C0003467 |
| Anxiety disorder | Unstructured | Condition occurrence | C0003469 |
| Anxiolytic drug therapy | All | Procedure occurrence | C4509951 |
| Anxiolytica | All | Drug exposure | C0078847, C0012010, C0078839, C0009011, C0009033, C0002333, C0024002 |
| Aripiprazol | All | Drug exposure | C0299792 |
| Asenapine | All | Drug exposure | C2000088 |
| Assisting with IADL | All | Procedure occurrence | C0516959 |
| Atrial fibrillation | All | Condition occurrence | C0004238 |
| Augmentation - atypical antipsychotics | All | Drug exposure | C0299792, C0073393, C0023870, C0123091, C0171023 |
| Auto-immune disease | All | Condition occurrence | C0004364 |
| Awareness of heart beat | Unstructured | Condition occurrence | C0476258 |
| Back Pain | Unstructured | Condition occurrence | C0004604 |
| Beck depression inventory score | All | Measurement | C2960571 |
| Bipolar disorder | All | Condition occurrence | C0005586 |
| Blood disorder | Unstructured | Condition occurrence | C0018939 |
| Blurred or darkened vision | Unstructured | Condition occurrence | C0344232 |
| BMI | Structured | Measurement | C1305855 |
| Borderline personality disorder | All | Condition occurrence | C0006012 |
| Brain stimulation | All | Procedure occurrence | C0870227 |
| Bromperidol | All | Drug exposure | C0054138 |
| Bulimia nervosa | All | Condition occurrence | C2267227 |
| Bupropion | All | Drug exposure | C0085208 |
| Cancer | All | Condition occurrence | C0006826 |
| Carbamazepine | All | Drug exposure | C0006949 |
| Cardiac arrhythmia | All | Condition occurrence | C0003811 |
| Cardiomyopathy | All | Condition occurrence | C0878544 |
| Cariprazine | All | Drug exposure | C2936870 |
| Central Nervous System Diseases | All | Condition occurrence | C0007682 |
| CGI | All | Measurement | LC5500037, C5396298, LC5500036, LC5500038 |
| CHA2DS2-VASc-score | All | Measurement | C4049268 |
| Charlson Comorbidity Index | All | Measurement | C4546361 |
| Chronic kidney disease | All | Condition occurrence | C1561643 |
| Circadian rhythm disorders | All | Condition occurrence | C0813142 |
| Citalopram | All | Drug exposure | C0008845 |
| Clomipramine | All | Drug exposure | C0009010 |
| Clotiapine | All | Drug exposure | C0009071 |
| Clozapine | All | Drug exposure | C0009079 |
| Cognitive behavioral analysis system of psychotherapy (CBASP) | All | Procedure occurrence | LC5500039 |
| Cognitive behavioural therapy | All | Procedure occurrence | C0009244 |
| Conduction disorder | All | Condition occurrence | C0264886 |
| Confusion | All | Condition occurrence | C0009676 |
| Connective tissue disorder | All | Condition occurrence | C3495801, C2347126, C0011644, C0008728, C0011633, C0003873, C0085655, C0009782 |
| COPD | All | Condition occurrence | C0024117 |
| Coronary artery disease | All | Condition occurrence | C1956346 |
| Corticosteroids | Structured | Drug exposure | C3540727, C0001617 |
| COVID-19 | All | Condition occurrence | C5203670 |
| Crisis intervention | All | Procedure occurrence | C0010332 |
| Crisis intervention for mental health | All | Procedure occurrence | C0204544, C1272285, C2551357 |
| CT | Structured | Procedure occurrence | C0040405 |
| CVS / Fibromyalgia | All | Condition occurrence | C0015674, C0016053 |
| Cystitis | Unstructured | Condition occurrence | C0010692 |
| Date of admission | Structured | Visit occurrence | C1302393 |
| Death | Structured | Person | C0011065 |
| Decrease in appetite | Unstructured | Condition occurrence | C0232462 |
| Dementia | All | Condition occurrence | C0497327 |
| Depressed mood | Unstructured | Condition occurrence | C0344315 |
| Depression | All | Condition occurrence | C0011581 |
| Depression management program | All | Procedure occurrence | C1303151 |
| Depressive disorder in remission | All | Measurement | C0544452 |
| Desire for urination | Unstructured | Measurement | C1321908 |
| Diabetes mellitus | All | Condition occurrence | C0011847, C0011849 |
| Diabetes type I | All | Condition occurrence | C0011854 |
| Diabetes type II | All | Condition occurrence | C0011860 |
| Dialysis | Structured | Procedure occurrence | C0011946 |
| Diarrhea | Unstructured | Condition occurrence | C0011991 |
| Diastolic blood pressure | Structured | Measurement | C0428883 |
| Discharge from hospital | Structured | Visit occurrence | C2361123 |
| Dissociative reaction | Unstructured | Condition occurrence | C0012746 |
| Divalproex | All | Drug exposure | C0042291 |
| Dizziness | Unstructured | Condition occurrence | C0012833 |
| Dosulepine | All | Drug exposure | C0013065 |
| Droperidol | All | Drug exposure | C0013136 |
| Drug abuse | All | Observation | C0013146 |
| Drug dependence | Unstructured | Condition occurrence | C1510472 |
| Drugs used for bipolar disorder | All | Drug exposure | C0023870, C0064636, C0123091, C0080356, C0006949 |
| Dry mouth | Unstructured | Condition occurrence | C0043352 |
| Duloxetine | All | Drug exposure | C0245561 |
| Dysgeusia | Unstructured | Measurement | C0013378 |
| Dyslipidemia | All | Condition occurrence | C0242339 |
| Dyspnea | All | Condition occurrence | C0013404 |
| Dyssomnias | All | Condition occurrence | C0700201 |
| Dysuria | Unstructured | Measurement | C0013428 |
| Electroconvulsive Therapy | All | Procedure occurrence | C0013806 |
| Episode of depression | All | Condition occurrence | C0349217 |
| Escitalopram | All | Drug exposure | C1099456 |
| Esketamine | All | Drug exposure | C2825616 |
| Family history of mental disorder | Unstructured | Measurement | C0455379 |
| Family problems | All | Measurement | C0424960 |
| Family therapy | All | Procedure occurrence | C0015618 |
| Fatigue | Unstructured | Condition occurrence | C0015672 |
| Fatigue | All | Condition occurrence | C0015672 |
| Feeling hopeless | Unstructured | Condition occurrence | C0150041 |
| Feeling nervous | Unstructured | Condition occurrence | C0849963 |
| Feeling upset | Unstructured | Condition occurrence | C0677661, C3887804 |
| Fever | All | Condition occurrence | C0015967 |
| Fibromyalgia | All | Condition occurrence | C0016053 |
| Finding of employment status | All | Measurement | C1287182 |
| Finding of level of interest | All | Measurement | C1287123 |
| Finding of marital or partnership status | All | Measurement | C1276230 |
| Finding of residence and accommodation circumstances | All | Measurement | C1268616 |
| Fluoxetine | All | Drug exposure | C0016365 |
| Flupentixol | All | Drug exposure | C0016367 |
| Fluspirileen | All | Drug exposure | C0016383 |
| Fluvoxamine | All | Drug exposure | C0085228 |
| Gastric ulcer | All | Condition occurrence | C0038358 |
| Glucose | Structured | Measurement | C0428554 |
| Haloperidol | All | Drug exposure | C0018546 |
| HAM-D | All | Measurement | C4545802 |
| Headache | Unstructured | Condition occurrence | C0018681 |
| Heart Diseases | All | Condition occurrence | C0018799 |
| Heart failure | All | Condition occurrence | C0018801, C1959583 |
| Height | Structured | Measurement | C0005890 |
| Hemiplegia | All | Condition occurrence | C0018991 |
| Highest level of education | All | Measurement | C4264309 |
| HIV | All | Condition occurrence | C0019693 |
| HIV1&2 Al/Ag | All | Measurement | C0485444 |
| Hostility | Unstructured | Condition occurrence | C0020039 |
| Household, family and support network finding | All | Measurement | C1287129 |
| Hypercholesterolemia | All | Condition occurrence | C0020443 |
| Hypersomnia | Unstructured | Condition occurrence | C0917799 |
| Hypertension | All | Condition occurrence | C0020538 |
| Hypoesthesia | Unstructured | Measurement | C0020580 |
| iADL | All | Measurement | C1290928, C0150641, C0518516 |
| Imipramine | All | Drug exposure | C0020934 |
| Improvement in level of depressed mood | All | Measurement | C5545418 |
| Increase in blood pressure | All | Measurement | C0497247 |
| Infection diseases | All | Condition occurrence | C0009450 |
| Interpersonal psychotherapy | All | Procedure occurrence | C0871787 |
| Involuntary admission | All | Procedure occurrence | C1277270 |
| Kidney Diseases | All | Condition occurrence | C0022658 |
| Lamotrigine | All | Drug exposure | C0064636 |
| Lethargy | Unstructured | Condition occurrence | C0023380 |
| Levomepromazine | All | Drug exposure | C0025678 |
| Lithium | All | Drug exposure | C0023870 |
| Liver diseases | All | Condition occurrence | C0577060, C0023895 |
| MADRS | All | Measurement | C4706358 |
| Major Depressive Disorder | All | Condition occurrence | C1269683 |
| Malignant neoplasm | All | Condition occurrence | C0006826 |
| MAO inhibitors | All | Drug exposure | C0026457 |
| Maprotiline | All | Drug exposure | C0024778 |
| Mental health team | All | Care site | C4300230 |
| Mianserine | All | Drug exposure | C0025912 |
| Mianserine | All | Drug exposure | C0025912 |
| Mild Major Depressive Disorder | All | Condition occurrence | C0270455 |
| Mindfulness | All | Procedure occurrence | C0556509 |
| Mirtazapine | All | Drug exposure | C0049506 |
| Moclobemide | All | Drug exposure | C0066673 |
| Moderate Major Depressive Disorder | All | Condition occurrence | C0270456 |
| Monoamineoxidase inhibitors | All | Drug exposure | C0066673 |
| More appetite or thirst | Unstructured | Measurement | C0241379, C0232461 |
| Mother tongue | All | Measurement | C0557072 |
| MRI | Structured | Procedure occurrence | C0024485 |
| Multiple sclerosis | All | Condition occurrence | C0026769 |
| Myocardial infarction | All | Condition occurrence | C0027051 |
| Myocarditis | All | Condition occurrence | C0027059 |
| Nausea | Unstructured | Condition occurrence | C0027497 |
| No response | All | Measurement | C0438286 |
| Noradrenaline and dopamine reuptake inhibitors | All | Drug exposure | C0085208 |
| Nortriptyline | All | Drug exposure | C0028420 |
| Nose dryness | Unstructured | Measurement | C0231919 |
| NSAID | Structured | Drug exposure | C0358845, C0003211 |
| NTproBNP | Structured | Measurement | C5217923, C1533071, C0754710 |
| Nycturia | Unstructured | Condition occurrence | C0028734 |
| Obesity | All | Condition occurrence | C0028754 |
| Obsessive-Compulsive Disorder | All | Condition occurrence | C0028768 |
| Obstructive sleep apnea | All | Condition occurrence | C0520679 |
| Olanzapine | All | Drug exposure | C0171023 |
| Organ transplant | All | Procedure occurrence | C0730400 |
| Osteoarthritis | All | Condition occurrence | C1384584, C0029408 |
| Pain | All | Condition occurrence | C0030193 |
| Paliperidon | All | Drug exposure | C0753678 |
| Palliative care | All | Procedure occurrence | C0030231 |
| Panic Attacks | Unstructured | Condition occurrence | C0086769 |
| Panic disorder | Unstructured | Condition occurrence | C0030319 |
| Parkinson's disease | All | Condition occurrence | C0030567 |
| Paroxetine | All | Drug exposure | C0070122 |
| Patient condition improved | All | Measurement | C0438108 |
| Patient condition worsened | All | Measurement | C1457868, C0438111, C1279889 |
| Patient cured | All | Measurement | C0438113 |
| Patient date of birth | Structured | Person | C0421451 |
| Patient sex | Structured | Person | C0079399 |
| Patient's condition stable | All | Observation | C0677946, C1282982 |
| Peptic ulcer | All | Condition occurrence | C0030920 |
| Peripheral arterial diseases | All | Condition occurrence | C1704436 |
| Peripheral vascular diseases | All | Condition occurrence | C0085096 |
| Personality disorder | All | Condition occurrence | C0031212 |
| Phenelzine | All | Drug exposure | C0031392 |
| PHQ-9 | All | Measurement | C4283755 |
| Pimozide | All | Drug exposure | C0031935 |
| Pipamperon | All | Drug exposure | C0071098 |
| Platelet count | Structured | Measurement | C1287267, C0032181 |
| Pollakisuria | Unstructured | Measurement | C0042023 |
| Post-traumatic stress disorder | Unstructured | Condition occurrence | C0038436 |
| Potassium | Structured | Measurement | C0428289 |
| Pregnant | All | Observation | C0033011, C0549206 |
| Problem solving therapy | All | Procedure occurrence | C1303140 |
| Prothipendyl | All | Drug exposure | C0139007 |
| Psychiatry clinic | All | Care site | C0020021, C3811913 |
| Psychiatry department | All | Care site | C0587494 |
| Psycho-analytic therapy | All | Procedure occurrence | C0392892, C0542300 |
| Psychodynamic psychotherapy | All | Procedure occurrence | C1261382 |
| Psychomotor retardation | Unstructured | Measurement | C5441816 |
| Psychotherapy | All | Procedure occurrence | C0033968 |
| Quetiapine | All | Drug exposure | C0123091 |
| Reboxetine | All | Drug exposure | C0168388 |
| Recurrent depression | All | Condition occurrence | C0221480 |
| Red blood cell count | Structured | Measurement | C1287262 |
| Relationship problems | All | Measurement | C0425168 |
| Relationship therapy | All | Procedure occurrence | C0730434 |
| Remission | All | Measurement | C0544452 |
| Renal dysfunction | All | Condition occurrence | C1565489 |
| Restlessness | Unstructured | Condition occurrence | C3887611 |
| Rheumatoid Arthritis | All | Condition occurrence | C0003873 |
| Risperidon | All | Drug exposure | C0073393 |
| RX | Structured | Procedure occurrence | C1306645 |
| Selective noradrenaline reuptake inhibitors | All | Drug exposure | C0168388 |
| Selective serotonin reuptake inhibitors (SSRI) | All | Drug exposure | C0074393, C0085228, C1099456, C0008845, C0070122, C0016365 |
| Serotonin and noradrenaline reuptake inhibitors | All | Drug exposure | C0245561, C0078569 |
| Sertindol | All | Drug exposure | C0084528 |
| Sertraline | All | Drug exposure | C0074393 |
| Severe Major Depressive Disorder | All | Condition occurrence | C3472470 |
| Sleeplessness | Unstructured | Condition occurrence | C0917801 |
| Sodium | Structured | Measurement | C0428291 |
| Spravato | All | Drug exposure | C4762645 |
| St John's wort | All | Drug exposure | C0813171 |
| Stomach diseases | All | Condition occurrence | C0038354 |
| Stroke / cerebrovascular accident (CVA) | All | Condition occurrence | C0038454 |
| Suicidal | Unstructured | Measurement | C0424000, C0038663 |
| Sulpiride | All | Drug exposure | C0038803 |
| Sweaty hands | Unstructured | Condition occurrence | C0424558 |
| Systolic blood pressure | Structured | Measurement | C0871470 |
| Throat irritation | Unstructured | Measurement | C0700184 |
| Thyroid disease | All | Condition occurrence | C0040128 |
| Tiapride | All | Drug exposure | C0040180 |
| Tobacco smoking status | All | Observation | C0453996 |
| Transcranial magnetic stimulation | All | Procedure occurrence | C0436548 |
| Transient ischemic attack (TIA) | All | Condition occurrence | C0007787 |
| Tranxene | All | Drug exposure | C0009033 |
| Trazodone | All | Drug exposure | C0040805 |
| Treatment Resistant Depression | All | Condition occurrence | C2063866 |
| Treatment response | All | Measurement | C0521982 |
| Tricyclic antidepressants | All | Drug exposure | C0013065, C0009010, C0028420, C0020934, C0002600 |
| Troponin level | Structured | Measurement | C0041199, C0523952, C5216123 |
| Valproate | All | Drug exposure | C0080356 |
| Venlafaxine | All | Drug exposure | C0078569 |
| Venous thromboembolism | All | Condition occurrence | C1861172 |
| Voluntary admission | All | Procedure occurrence | C0376348 |
| Vomiting | All | Condition occurrence | C0042963 |
| Vortioxetine | All | Drug exposure | C3661282 |
| Weight | Structured | Measurement | C0005910 |
| Weight gain | Unstructured | Condition occurrence | C0043094 |
| Weight loss | Unstructured | Condition occurrence | C1262477 |
| White blood cell count | Structured | Measurement | C0427512 |
| Zuclopenthixol | All | Drug exposure | C0376160 |
| Zung (ZSDS) | All | Measurement | C4273947 |

**Supplementary Table 3.** NLP pipeline performance metrics

| **Datapoint** | **Precision, OOTB (%)** | **Precision, mitigated (%)** | **Precision (n)** | **Recall, OOTB (%)** | **Recall, mitigated (%)** | **Recall (n)** | **F1 score (%)** |
| --- | --- | --- | --- | --- | --- | --- | --- |
| Amisulpride | 100.0 | 100.0 | 2 | NA | NA |  | NA |
| Amitriptyline | NA | NA | 0 | NA | 100.0 | 1 | NA |
| Anhedonia | 100.0 | 100.0 | 5 | 92.3 | 100.0 | 13 | 100.0 |
| Anorexia nervosa | NA | NA | 0 | 100.0 | 100.0 | 1 | NA |
| Antidepressants | 100.0 | 100.0 | 16 | 74.5 | 100.0 | 47 | 100.0 |
| Antidepressants acting on neuroreceptors | 85.7 | 100.0 | 7 | 90.0 | 100.0 | 10 | 100.0 |
| Antipsychotics | 86.2 | 100.0 | 65 | 66.7 | 100.0 | 9 | 100.0 |
| Anxiety | 95.2 | 100.0 | 42 | 42.4 | 93.9 | 33 | 96.9 |
| Anxiety disorder | 90.8 | 97.4 | 76 | 100.0 | 100.0 | 14 | 98.7 |
| Anxiolytica | 81.3 | 93.8 | 16 | 88.9 | 100.0 | 9 | 96.8 |
| Aripiprazol | 75.0 | 100.0 | 4 | NA | NA | 0 | NA |
| Augmentation - atypical antipsychotics | 100.0 | 100.0 | 11 | 57.1 | 100.0 | 7 | 100.0 |
| Back Pain | 100.0 | 100.0 | 2 | NA | 100.0 | 1 | 100.0 |
| Bipolar disorder | 83.3 | 100.0 | 6 | 33.3 | 100.0 | 3 | 100.0 |
| Borderline personality disorder | 84.0 | 96.0 | 50 | NA | 100.0 | 1 | 98.0 |
| Bupropion | 100.0 | 100.0 | 2 | 100.0 | 100.0 | 3 | 100.0 |
| Central Nervous System Diseases | 94.1 | 100.0 | 17 | 57.1 | 100.0 | 7 | 100.0 |
| Circadian rhythm disorders | NA | NA | 0 | NA | 100.0 | 1 | NA |
| Clotiapine | 100.0 | 100.0 | 1 | 100.0 | 100.0 | 2 | 100.0 |
| Crisis intervention | 82.0 | 92.0 | 50 | NA | 100.0 | 1 | 95.8 |
| Crisis intervention for mental health | 100.0 | 100.0 | 1 | NA | 100.0 | 2 | 100.0 |
| Cystitis | 100.0 | 100.0 | 2 | NA | 100.0 | 2 | 100.0 |
| Decrease in appetite | 100.0 | 100.0 | 1 | 75.0 | 100.0 | 7 | 100.0 |
| Dementia | 86.5 | 92.3 | 52 | NA | 100.0 | 6 | 96.0 |
| Depressed mood | 97.7 | 97.7 | 44 | 39.3 | 95.2 | 84 | 96.5 |
| Depression | 88.5 | 97.4 | 78 | 58.8 | 96.1 | 51 | 96.8 |
| Diabetes mellitus | 79.8 | 94.6 | 223 | 97.2 | 97.9 | 95 | 96.2 |
| Diarrhea | 84.6 | 92.3 | 13 | 100.0 | 100.0 | 31 | 96.0 |
| Dizziness | 91.2 | 97.1 | 102 | 90.0 | 100.0 | 14 | 98.5 |
| Drug dependence | 66.7 | 100.0 | 3 | 57.1 | 100.0 | 7 | 100.0 |
| Drugs used for bipolar disorder | 100.0 | 100.0 | 9 | 75.0 | 100.0 | 6 | 100.0 |
| Dry mouth | 100.0 | 100.0 | 1 | NA | NA | 0 | NA |
| Duloxetine | 80.0 | 100.0 | 5 | 50.0 | 100.0 | 8 | 100.0 |
| Dyssomnias | 100.0 | 100.0 | 7 | 69.2 | 100.0 | 13 | 100.0 |
| Dysuria | 100.0 | 100.0 | 4 | 50.0 | 100.0 | 5 | 100.0 |
| Electroconvulsive Therapy | 92.7 | 98.2 | 55 | 93.8 | 100.0 | 16 | 99.1 |
| Episode of depression | 100.0 | 100.0 | 5 | 100.0 | 100.0 | 6 | 100.0 |
| Escitalopram | 100.0 | 100.0 | 3 | 100.0 | 100.0 | 2 | 100.0 |
| Family history of mental disorder | NA | NA | 0 | NA | 100.0 | 3 | NA |
| Family problems | 100.0 | 100.0 | 1 | NA | 100.0 | 3 | 100.0 |
| Fatigue | 80.0 | 95.0 | 20 | 73.9 | 97.1 | 68 | 96.0 |
| Feeling hopeless | NA | NA | 0 | 66.7 | 100.0 | 6 | NA |
| Feeling upset | NA | NA | 0 | NA | 100.0 | 1 | NA |
| Finding of employment status | 57.1 | 100.0 | 7 | 33.3 | 100.0 | 9 | 100.0 |
| Finding of level of interest | 100.0 | 100.0 | 1 | 50.0 | 100.0 | 2 | 100.0 |
| Finding of marital or parternship status | 91.7 | 91.7 | 12 | 66.7 | 100.0 | 6 | 95.7 |
| Finding of residence and accommodation circumstances | 75.0 | 100.0 | 4 | NA | 100.0 | 3 | 100.0 |
| Fluoxetine | NA | NA | 0 | 66.7 | 100.0 | 3 | NA |
| Headache | 100.0 | 100.0 | 9 | 94.4 | 100.0 | 21 | 100.0 |
| Heart Diseases | 97.2 | 98.6 | 71 | 50.0 | 100.0 | 7 | 99.3 |
| Highest level of education | NA | NA | 0 | NA | 100.0 | 2 | NA |
| Household, family and support network finding | 73.5 | 94.1 | 34 | 37.5 | 91.7 | 24 | 92.9 |
| Hypertension | 96.3 | 98.1 | 215 | 91.0 | 98.4 | 125 | 98.3 |
| iADL | 100.0 | 100.0 | 1 | NA | 100.0 | 1 | 100.0 |
| Improvement in level of depressed mood | NA | NA | 0 | NA | 100.0 | 1 | NA |
| Infection diseases | 50.0 | 100.0 | 4 | NA | NA | 0 | NA |
| Involuntary admission | 74.1 | 98.1 | 54 | NA | 100.0 | 3 | 99.1 |
| Kidney diseases | 97.1 | 98.6 | 69 | 86.2 | 97.6 | 164 | 98.1 |
| Lamotrigine | NA | NA | 0 | 100.0 | 100.0 | 1 | NA |
| Lithium | 100.0 | 100.0 | 1 | 100.0 | 100.0 | 1 | 100.0 |
| Liver diseases | 85.4 | 97.6 | 41 | 73.7 | 95.3 | 43 | 96.4 |
| Lung diseases | 84.6 | 95.3 | 214 | 61.9 | 98.4 | 126 | 96.8 |
| Major Depressive Disorder | 100.0 | 100.0 | 7 | 10.0 | 95.0 | 20 | 97.4 |
| Malignant neoplasm | 76.1 | 90.1 | 71 | 100.0 | 100.0 | 2 | 94.8 |
| Mental Health Assessment | 100.0 | 100.0 | 9 | 100.0 | 100.0 | 11 | 100.0 |
| Mindfullness | 100.0 | 100.0 | 1 | NA | NA | 0 | NA |
| Mirtazapine | 98.1 | 100.0 | 53 | 100.0 | 100.0 | 4 | 100.0 |
| Moclobemide | NA | NA | 0 | 66.7 | 100.0 | 3 | NA |
| Monoamineoxidase inhibitors | NA | NA | 0 | 66.7 | 100.0 | 3 | NA |
| Nausea | 86.2 | 100.0 | 65 | 100.0 | 97.7 | 44 | 98.9 |
| No response | NA | NA | 0 | NA | 100.0 | 1 | NA |
| Noradrenaline and dopamine reuptake inhibitors | 100.0 | 100.0 | 1 | 100.0 | 100.0 | 3 | 100.0 |
| Obesity | NA | NA | 0 | 100.0 | 100.0 | 4 | NA |
| Olanzapine | 66.7 | 66.7 | 3 | 100.0 | 100.0 | 1 | 80.0 |
| Paliperidon | 100.0 | 100.0 | 1 | NA | NA | 0 | NA |
| Panic Attacks | 98.1 | 100.0 | 54 | 100.0 | 100.0 | 4 | 100.0 |
| Parkinson's disease | 100.0 | 100.0 | 1 | NA | NA | 0 | NA |
| Paroxetine | 100.0 | 100.0 | 1 | 100.0 | 100.0 | 1 | 100.0 |
| Patient condition improved | NA | 100.0 | 1 | NA | 100.0 | 3 | 100.0 |
| Patient condition worsened | NA | NA | 0 | NA | 100.0 | 1 | NA |
| Personality disorder | 50.0 | 100.0 | 6 | 60.0 | 100.0 | 6 | 100.0 |
| Phenelzine | 100.0 | 100.0 | 1 | NA | 100.0 | 5 | 100.0 |
| Post-traumatic stress disorder | 84.0 | 94.0 | 50 | 100.0 | 100.0 | 1 | 96.9 |
| Prothipendyl | 100.0 | 100.0 | 2 | 100.0 | 100.0 | 2 | 100.0 |
| Psychiatry clinic | 92.3 | 92.3 | 13 | 16.7 | 100.0 | 11 | 96.0 |
| Psychiatry department | 90.5 | 90.5 | 21 | 100.0 | 100.0 | 18 | 95.0 |
| Psychotherapy | 85.7 | 100.0 | 7 | 83.3 | 100.0 | 8 | 100.0 |
| Quetiapine | 100.0 | 100.0 | 1 | 50.0 | 100.0 | 4 | 100.0 |
| Recurrent depression | 86.3 | 100.0 | 51 | NA | 100.0 | 4 | 100.0 |
| Relationship problems | 100.0 | 100.0 | 3 | 40.0 | 100.0 | 5 | 100.0 |
| Restlessness | 100.0 | 100.0 | 5 | NA | 100.0 | 2 | 100.0 |
| Rheumatoid arthritis | 98.5 | 100.0 | 137 | 83.0 | 94.3 | 53 | 97.1 |
| Risperidon | NA | NA | 0 | 100.0 | 100.0 | 1 | NA |
| Selective serotonin reuptake inhibitors (SSRI) | 100.0 | 100.0 | 59 | 88.9 | 100.0 | 9 | 100.0 |
| Seretonin and noradrenaline reuptake inhibitors | 98.2 | 98.2 | 55 | 73.3 | 100.0 | 15 | 99.1 |
| Sertraline | 100.0 | 100.0 | 5 | 100.0 | 100.0 | 3 | 100.0 |
| Severe Major Depressive Disorder | 87.0 | 96.3 | 54 | NA | 100.0 | 8 | 98.1 |
| Sleeplessness | 100.0 | 100.0 | 4 | 75.0 | 100.0 | 12 | 100.0 |
| Stomach diseases | 100.0 | 100.0 | 1 | NA | NA | 0 | NA |
| Stroke / cerebrovascular accident (CVA) | 85.7 | 90.5 | 105 | 92.9 | 100.0 | 40 | 95.0 |
| Suicidal | 89.2 | 90.3 | 93 | 35.4 | 95.8 | 48 | 93.0 |
| Thyroid disease | 100.0 | 100.0 | 2 | NA | NA | 0 | NA |
| Transcranial magnetic stimulation | NA | NA | 0 | NA | 100.0 | 1 | NA |
| Trazodone | 100.0 | 100.0 | 2 | 100.0 | 100.0 | 6 | 100.0 |
| Treatment Resistant Depression | 92.9 | 100.0 | 14 | NA | 100.0 | 3 | 100.0 |
| Treatment response | 66.9 | 97.3 | 149 | 68.0 | 98.1 | 53 | 97.7 |
| Tricyclic antidepressants | 100.0 | 100.0 | 1 | NA | 100.0 | 1 | 100.0 |
| Valproate | 100.0 | 100.0 | 1 | NA | NA | 0 | NA |
| Venlafaxine | 100.0 | 100.0 | 4 | 100.0 | 100.0 | 6 | 100.0 |
| Weight gain | NA | NA | 0 | NA | 100.0 | 1 | NA |
| Weight loss | 42.4 | 90.9 | 33 | 85.7 | 100.0 | 21 | 95.2 |
| **Total** | **Mean = 87.5** | **Mean = 96.6** | **N = 2885** | **Mean = 72.3** | **Mean = 98.1** | **N = 1627** | **Mean = 98.5** |
